# Supplementary material for: Development of Improved Spectrophotometric Assays for Biocatalytic Silyl Ether Hydrolysis
Source: Biomolecules. 2024 Apr 18;14(4):492. doi: 10.3390/biom14040492 (PMC11048244; doi:10.3390/biom14040492)
Supplement: Supplementary file 1 [file biomolecules-14-00492-s001.zip › biomolecules-2937218-supplementary.pdf]

Supplementary Information

Development of Improved Spectrophotometric Assays for Biocatalytic Silyl Ether Hydrolysis

Yuqing Lu [1,2,†] Chisom S. Egedeuzu [1,2,†] Peter G. Taylor [3] and Lu Shin Wong [1,2,*]

[1] Manchester Institute of Biotechnology, University of Manchester, Manchester M1 7DN, UK; yuqing.lu-3@postgrad.manchester.ac.uk (Y.L.); chisom.egedeuzu@manchester.ac.uk (C.S.E.)

[2] Department of Chemistry, University of Manchester, Manchester M13 9PL, UK

[3] School of Life Health and Chemical Sciences, Open University, Milton Keynes MK7 6AA, UK; peter.taylor@open.ac.uk

* Correspondence: l.s.wong@manchester.ac.uk

† These authors contributed equally to the work.

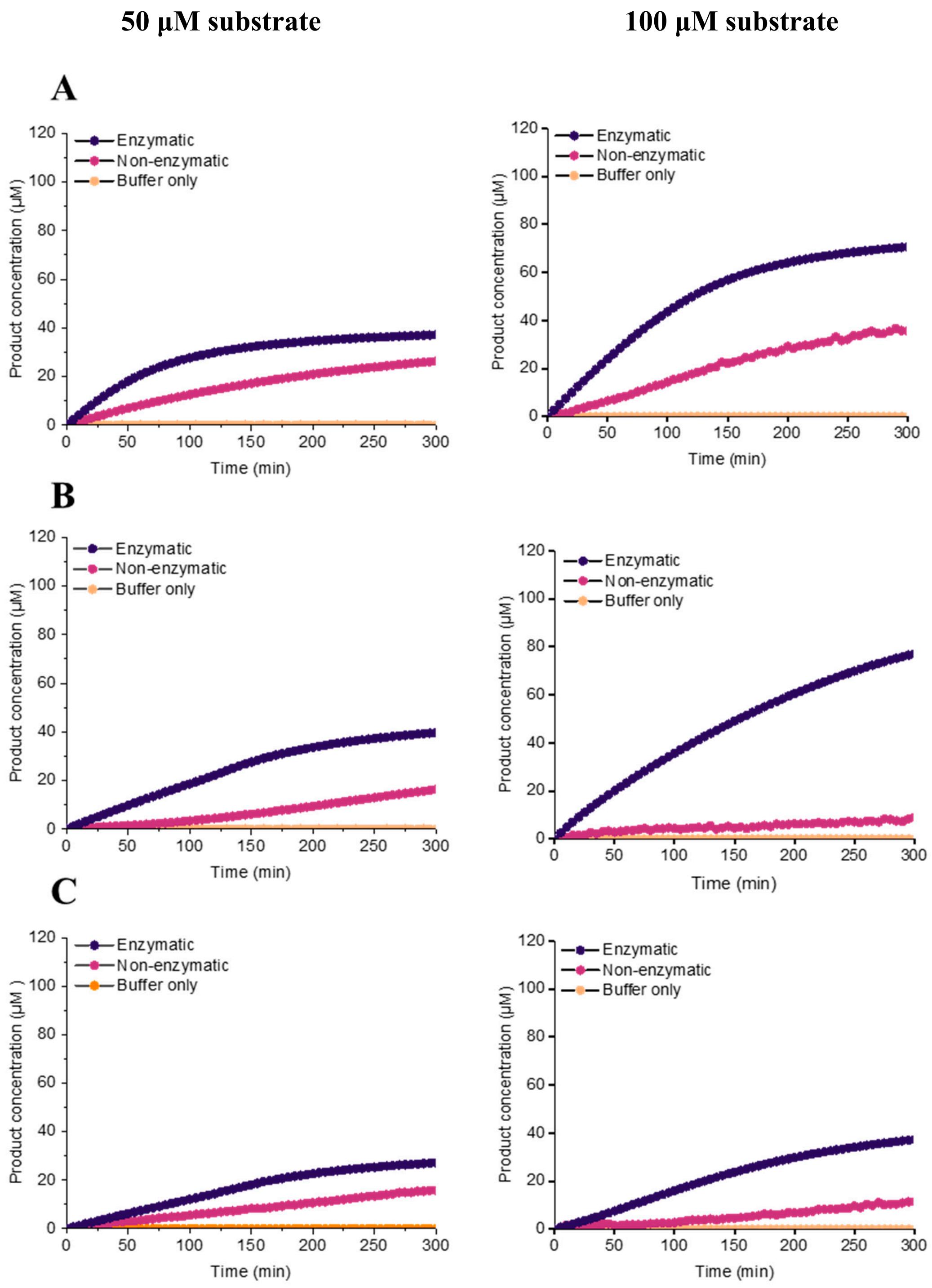

Figure S1. Graph of concentration of nitrophenolate produced against time for enzymatic and non-enzymatic hydrolysis measured at the λ_{max} of their corresponding 4-nitrophenolate ions. (**A**) substrate **1** at 405 nm, (**B**) substrate **4** at 414 nm, (**C**) substrate **5** at 394 nm, (**D**, next page) substrate **6** at 398 nm, (**E**) substrate **7** at 294 nm. Data were quantified using their corresponding calibration curves (Supporting Information Figures S4). Reactions were carried out with 6.7 μM TF-Silα-Strep, 50 μM substrate (Left), 100 μM substrate (Right), 10 % v/v 1,4-dioxane, 50 mM Tris buffer at the pH 8.5and 100 mM NaCl.

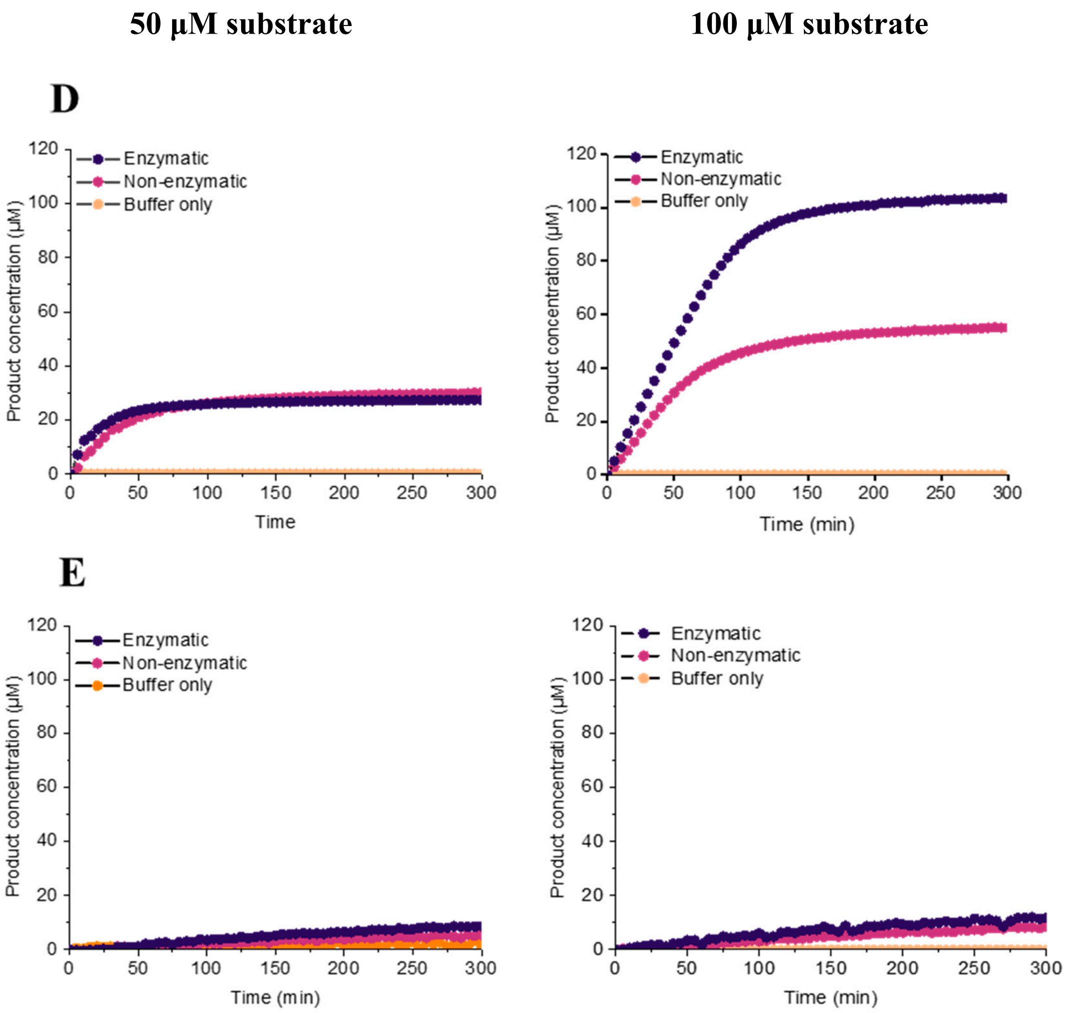

Figure S1 (continued). Graph of concentration of silanols produced showing enzymatic and non-enzymatic (background) hydrolysis measured at the λ_{max} of their corresponding 4-nitrophenolate ions after 300 min. Buffer only is used as the blank. (**A**) substrate **1** at 405 nm, (**B**) substrate **4** at 414 nm, (**C**) substrate **5** at 394 nm, (**D**, next page) substrate **6** at 398 nm, (**E**) substrate **7** at 294 nm. Data were quantified using their corresponding calibration curves (Supporting Information Figures S4). Reactions were carried out with 6.7 µM TF-Silα-Strep, 50 µM substrate (Left), 100 µM substrate (Right), 10 % v/v 1,4-dioxane, 50 mM Tris buffer at the pH 8.5and 100 mM NaCl.

Table S1. Rates of enzymatic *vs*. background hydrolysis showing the initial rates and fold increase of enzyme-catalysed hydrolysis of different silyl ether substrates. Enzymatic reactions were carried out with 6.7 µM TF-Silα-Strep, 50 or 100 µM substrate, 10 % v/v 1,4-dioxane, 50 mM Tris buffer at pH 8.5 and 100 mM NaCl.

Substrate	Initial rate at 50 µM substrate (µM min^{-1})			Fold difference†	Initial rate at 100 µM substrate (µM min^{-1})			Fold difference†
	Enzymatic	Background	Net*		Enzymatic	Background	Net*	
**1**	0.44	0.15	0.29	2.9	0.80	0.37	0.43	2.2
**4**	0.19	0.03	0.16	6.2	0.35	0.03	0.32	11.6
**5**	0.12	0.06	0.06	2.0	0.14	0.05	0.09	2.8
**6**	0.72	0.54	0.18	1.3	1.36	0.79	0.57	1.7
**7**	0.04	0.027	0.013	1.5	0.04	0.03	0.01	1.3

*Net initial rate is measured as the difference in initial rate between the enzymatic and background hydrolysis.

†Fold difference (rate ratio) calculated as the ratio of the enzymatic reaction relative to background (non-enzymatic) reaction.

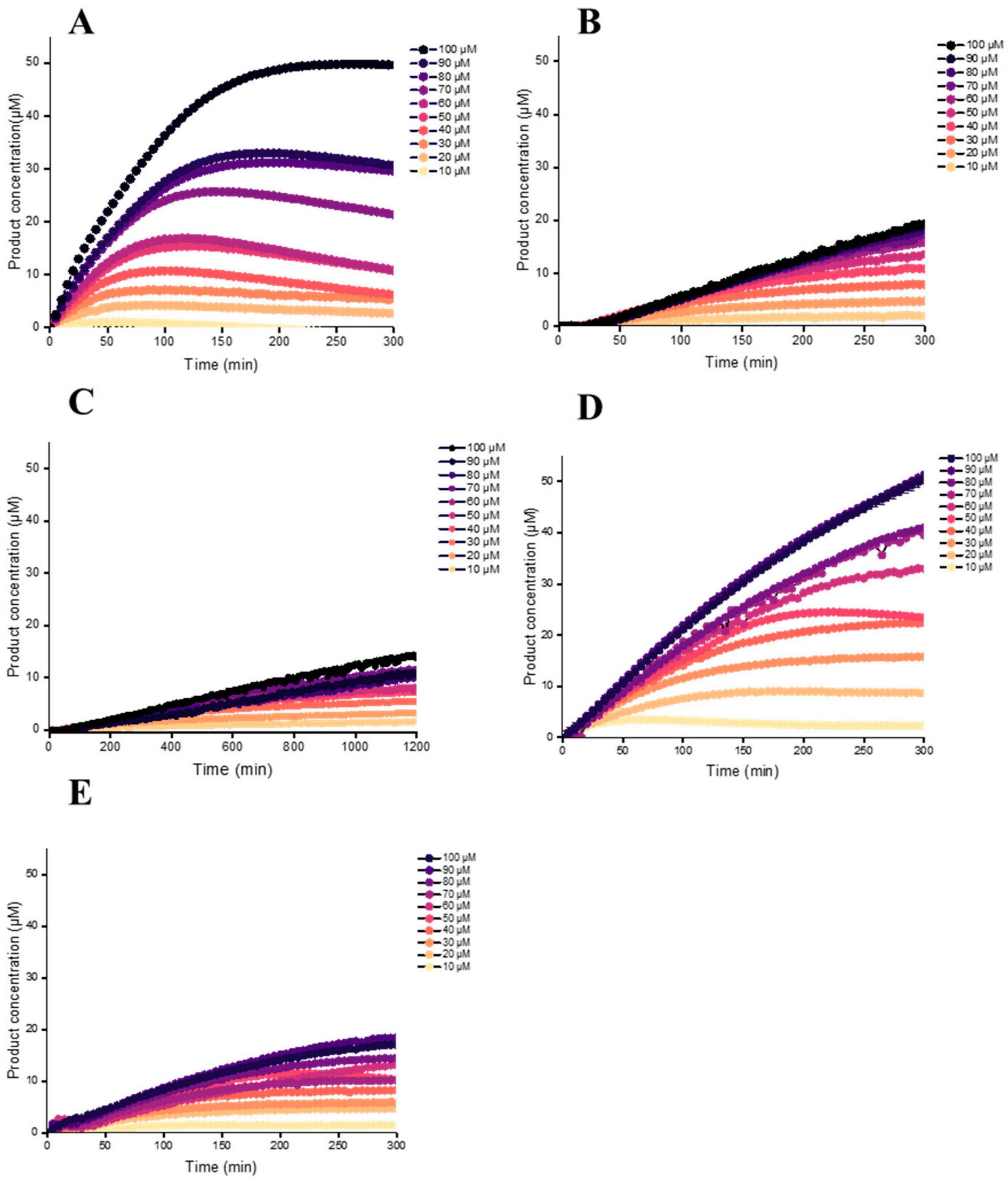

Figure S2. Graph of net concentration of corresponding phenoxide products measured by UV-Vis absorbance at their respective λ_{max}**.** Substrate **1** (**A**), **2** (**B**), and **3** (**C**) at 405 nm; **4** (**D**) at 414 nm and **5** (**E**) at 394 nm. Data were calibrated using data from Supporting Information Figures S4, and normalised with respect to the x- and y-axes.

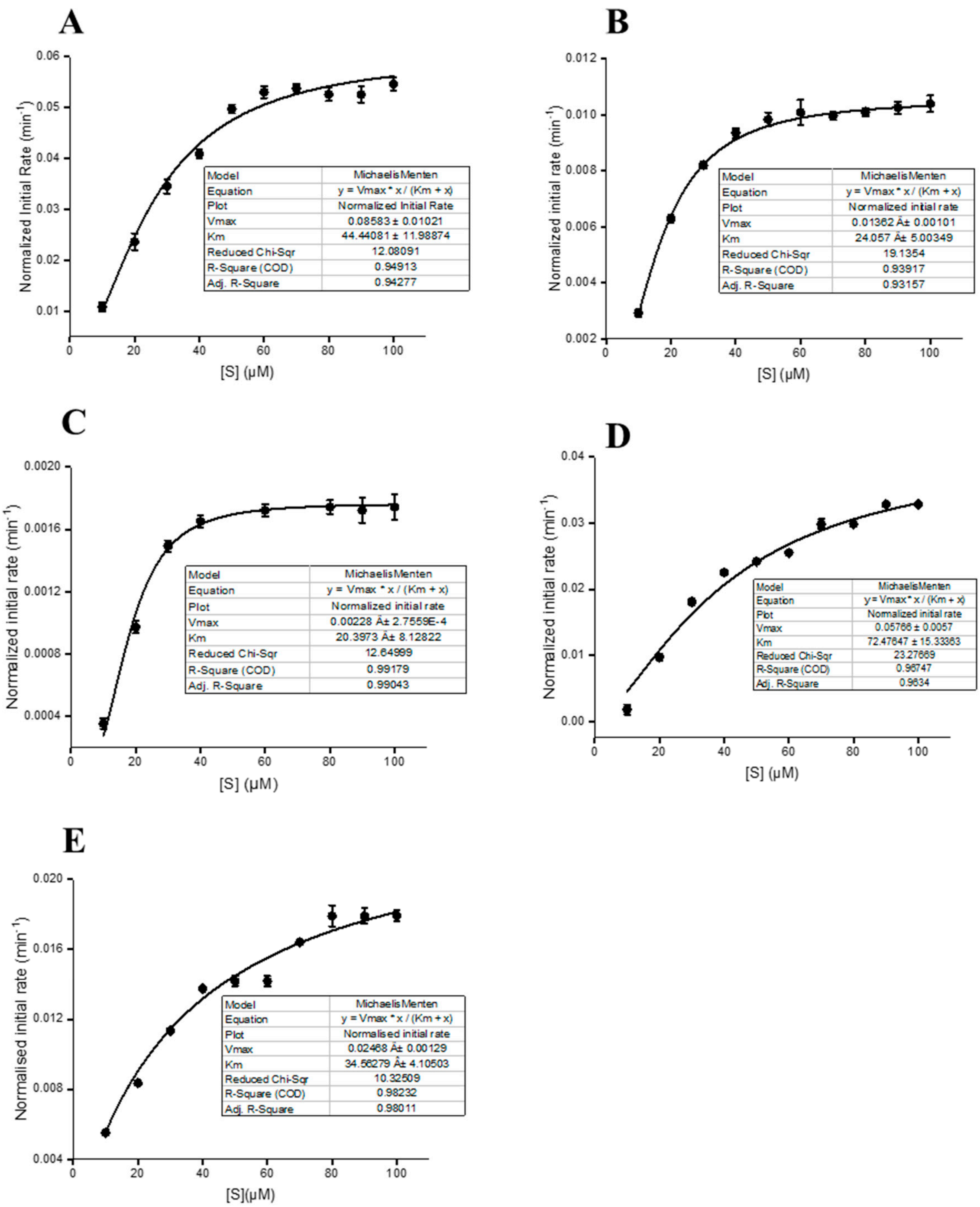

Figure S3. Graph of best fit Michaelis–Menten curves for the hydrolysis of silyl ether substrates by TF-Silα-Strep against a range of substrate concentrations. Substrate **1** (**A**), **2** (**B**), **3** (**C**), **4** (**D**) and **5** (**E**).

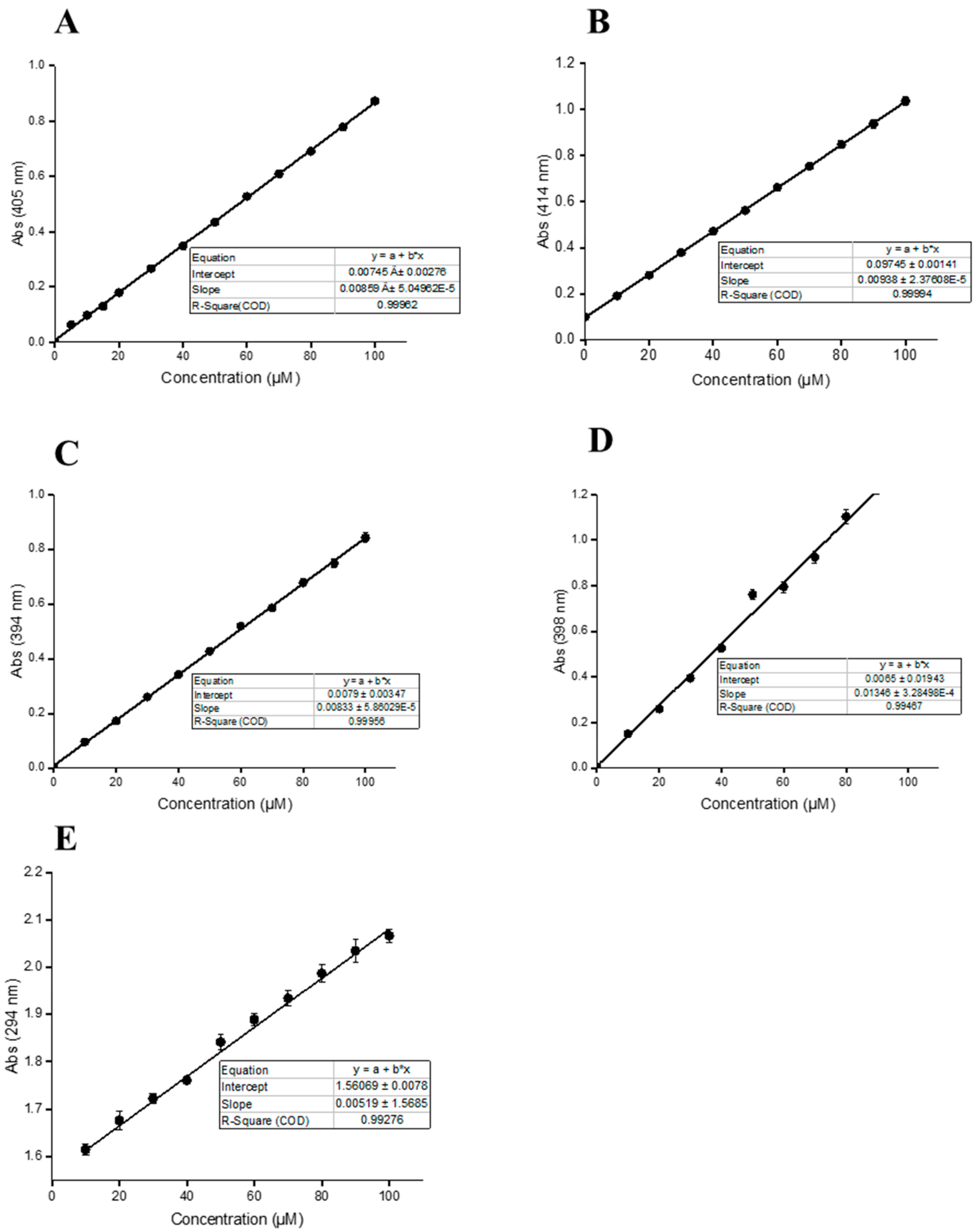

Figure S4. Calibration graph of UV-Vis absorption against concentration of (**A**) 4-nitrophenol for **1**, (**B**) 2-methyl-4-nitrophenol for **4**, (**C**) 3-methyl-4-nitrophenol for **5**, (**D**) 3-methoxy-4-nitrophenol for **6**, (**E**) 4-cyanophenol for **7**, in buffer (Tris (50 mM), NaCl (100 mM), pH 8.5, 10% (v/v) 1,4-dioxane).

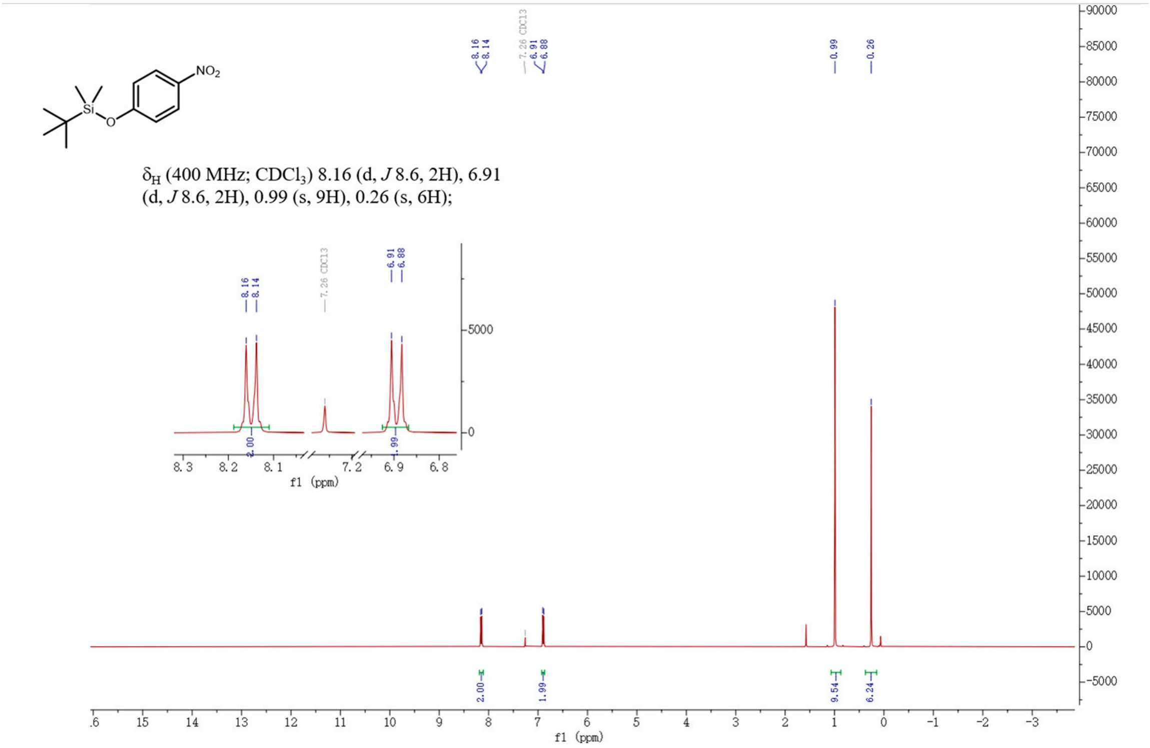

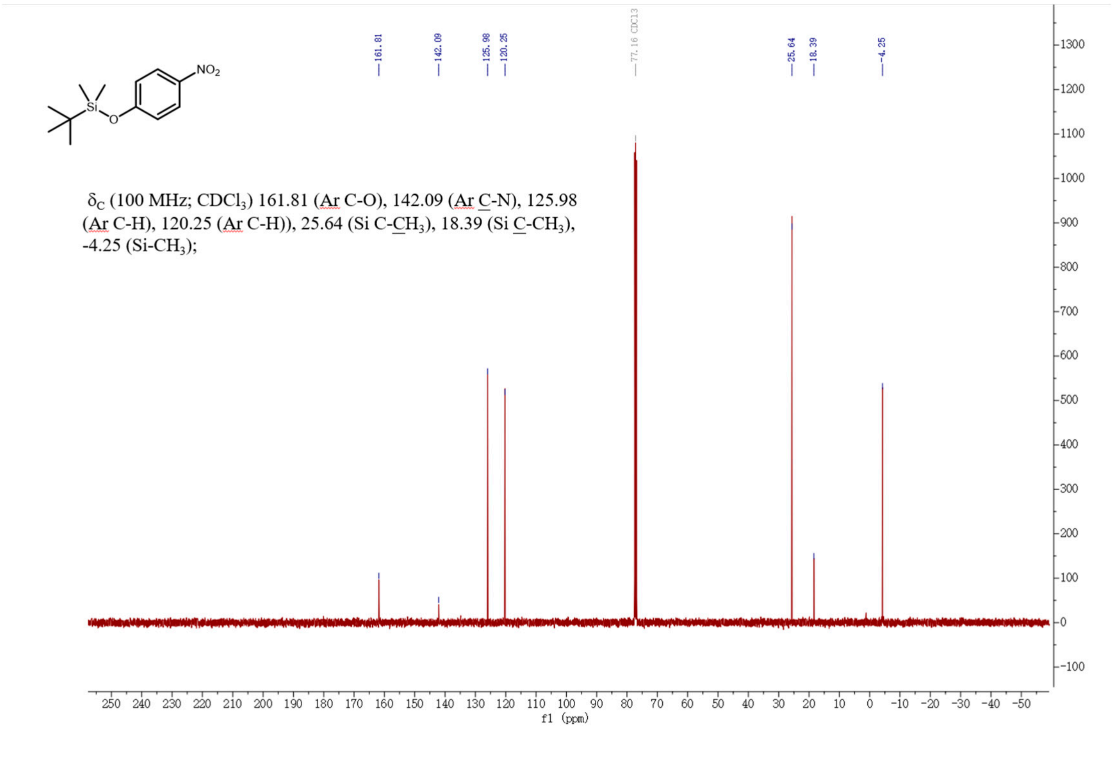

Figure S5. Calibrated NMR spectra for **1** showing ^{1}H (**top**) and ^{13}C (**bottom**) chemical shifts.

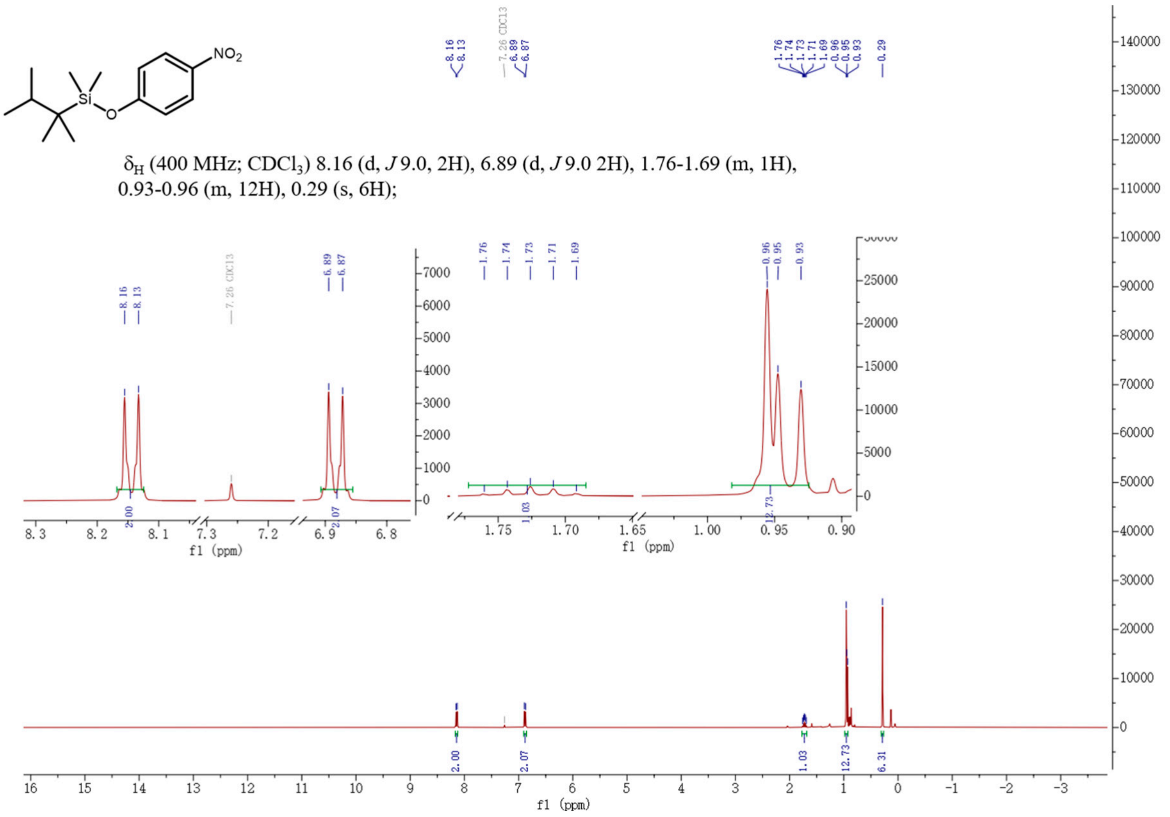

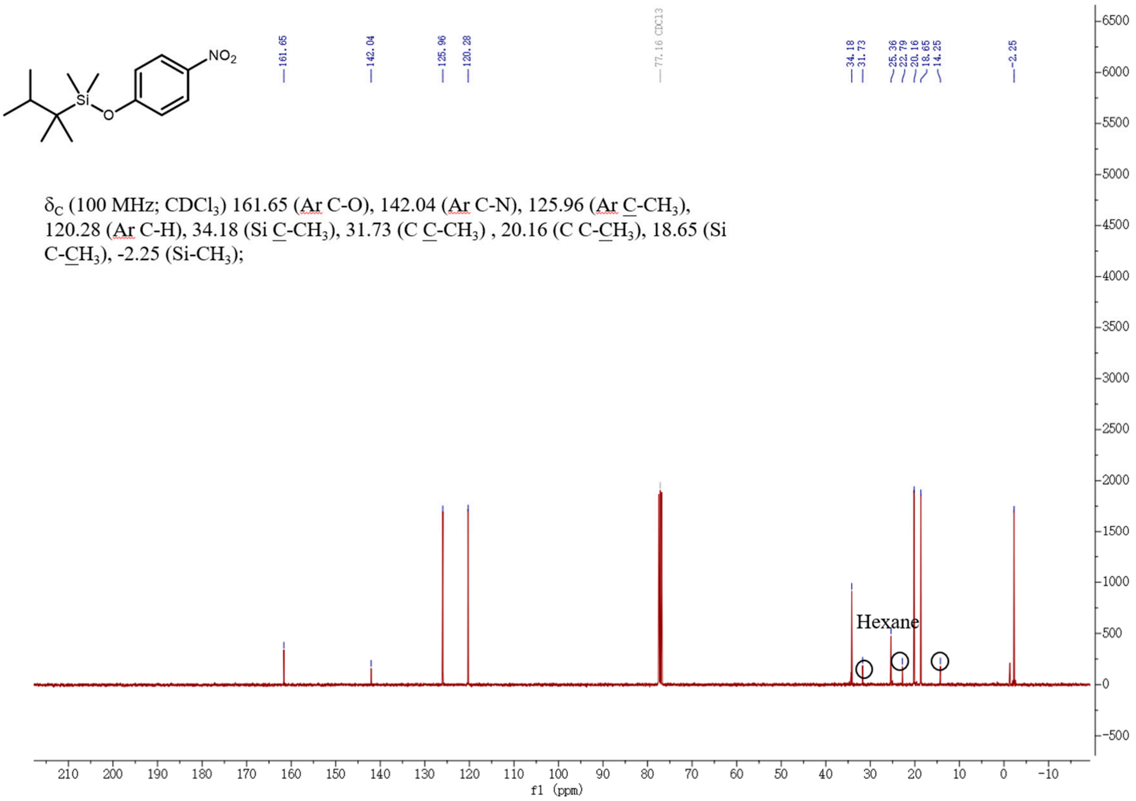

Figure S6. Calibrated NMR spectra for **2** showing ^{1}H (**top**) and ^{13}C (**bottom**) chemical shifts.

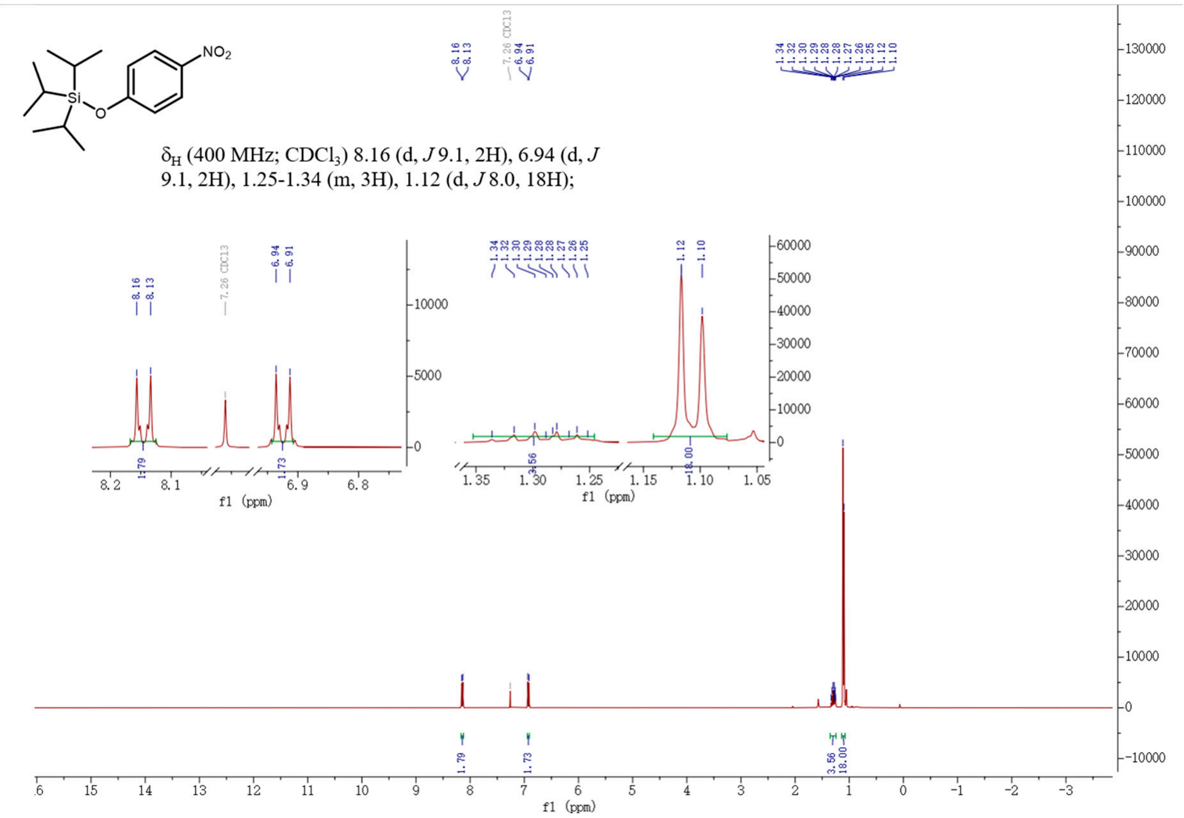

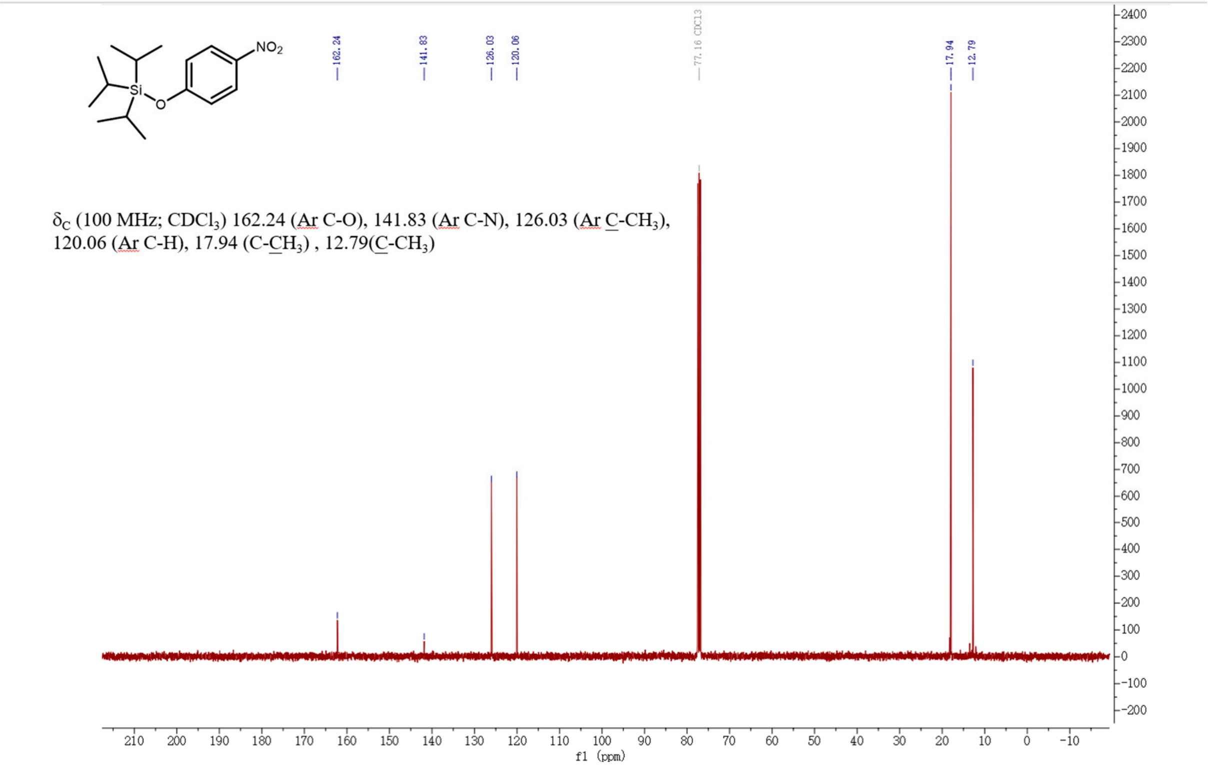

Figure S7. Calibrated NMR spectra for **3** showing ^{1}H (**top**) and ^{13}C (**bottom**) chemical shifts.

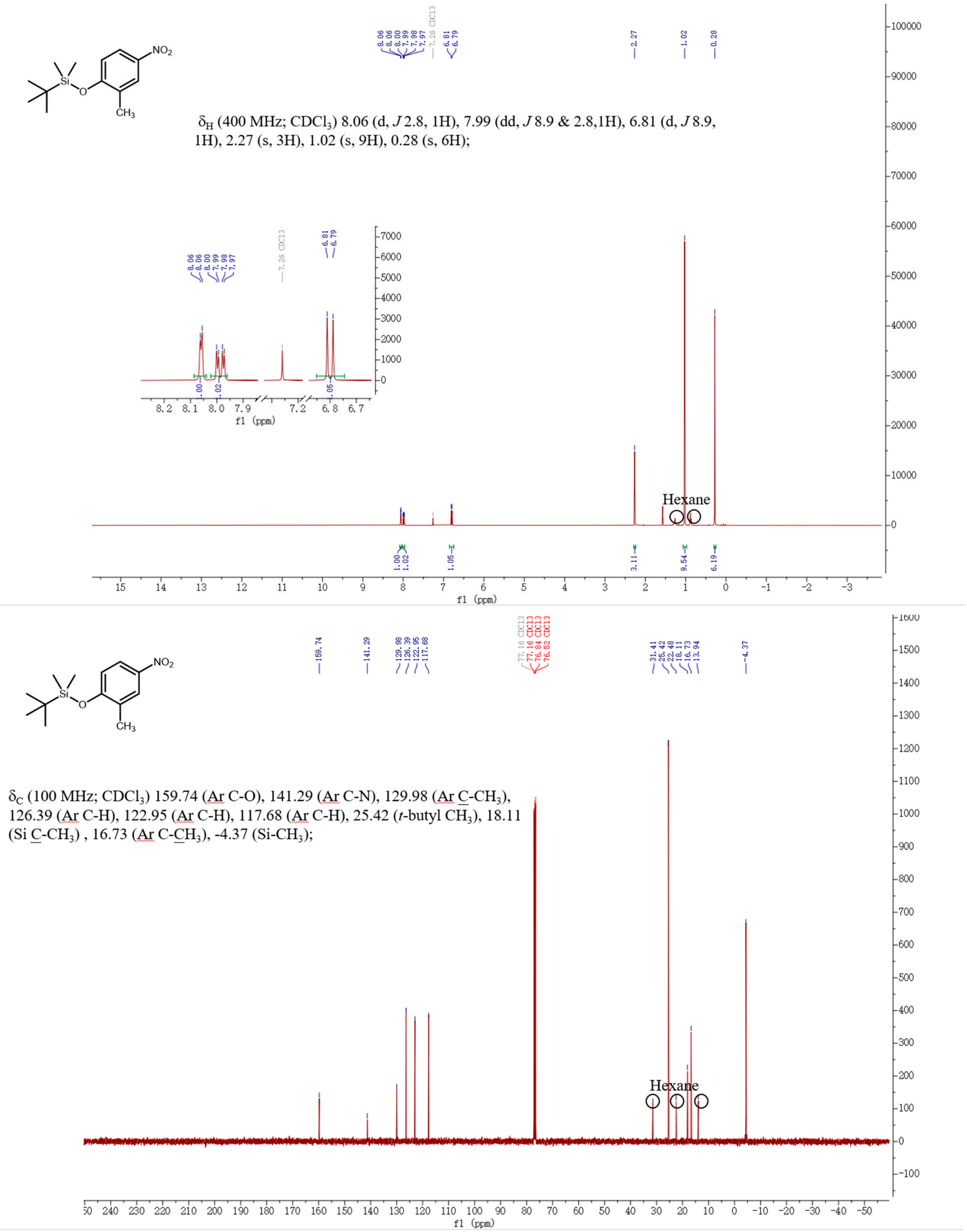

Figure S8. Calibrated NMR spectra for **4** showing ^{1}H (**top**) and ^{13}C (**bottom**) chemical shifts.

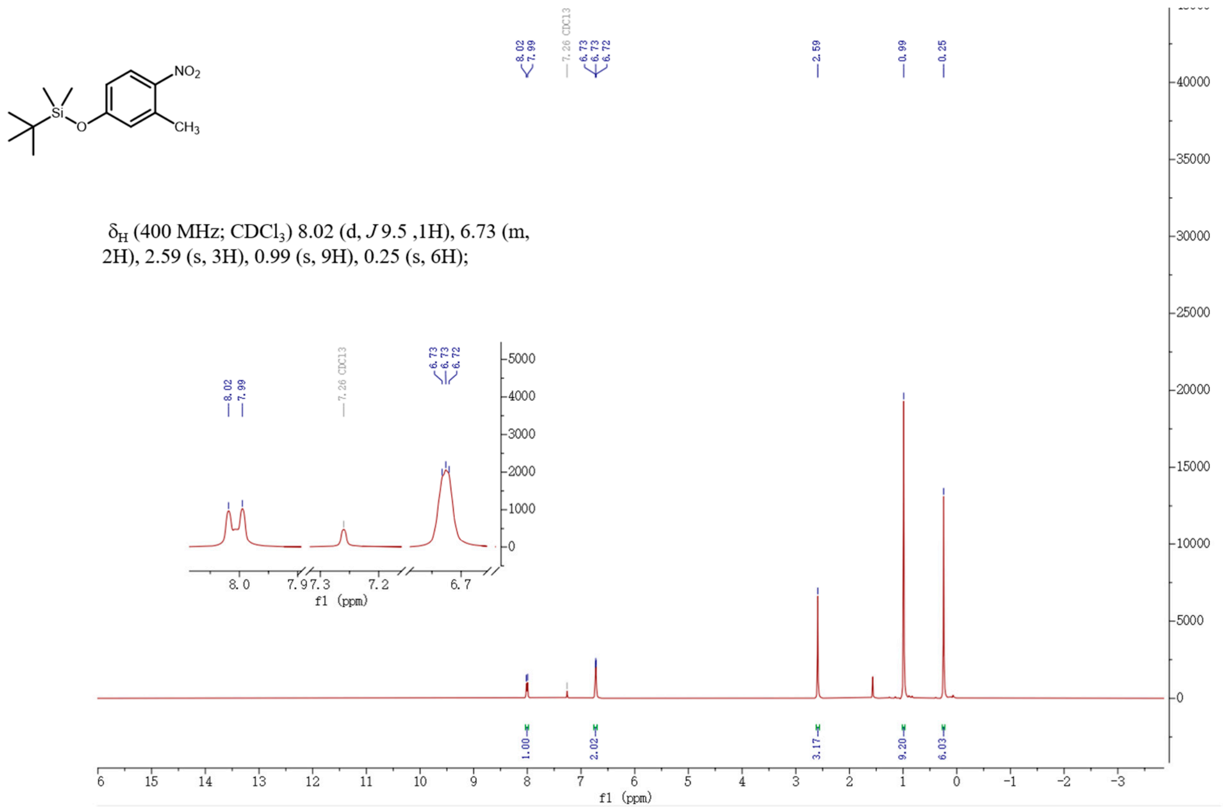

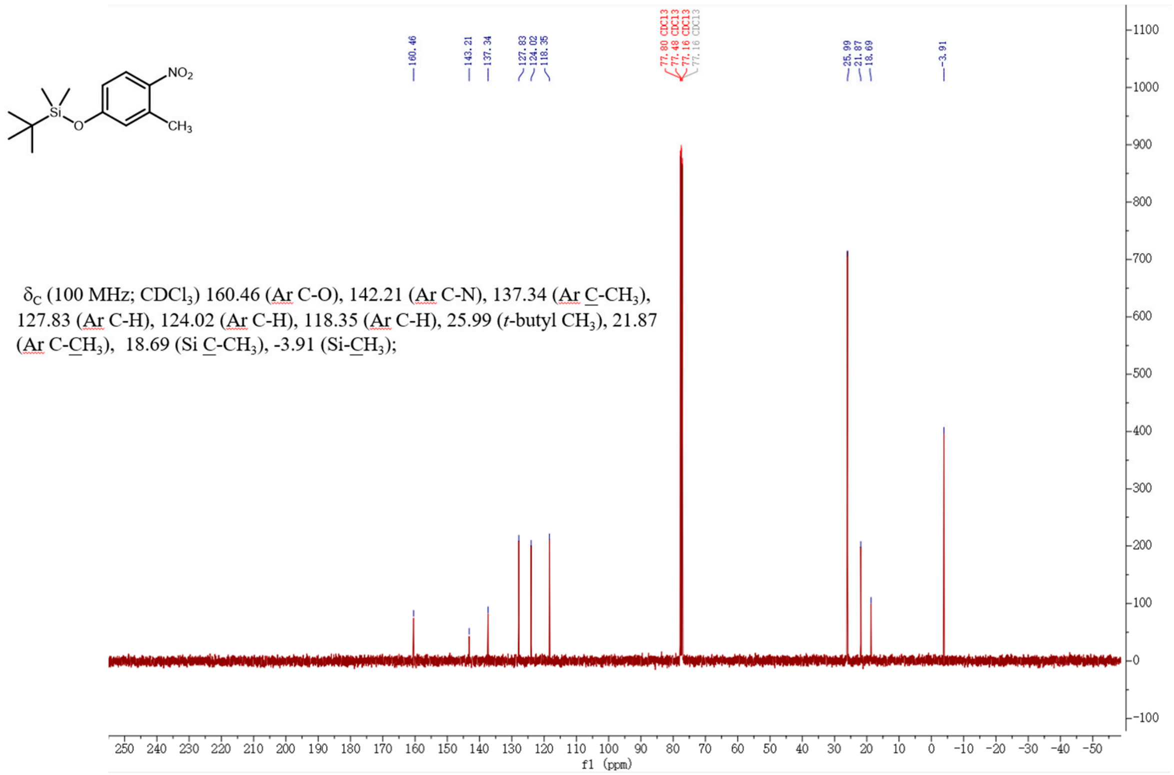

Figure S9. Calibrated NMR spectra for **5** showing ^{1}H (**top**) and ^{13}C (**bottom**) chemical shifts.

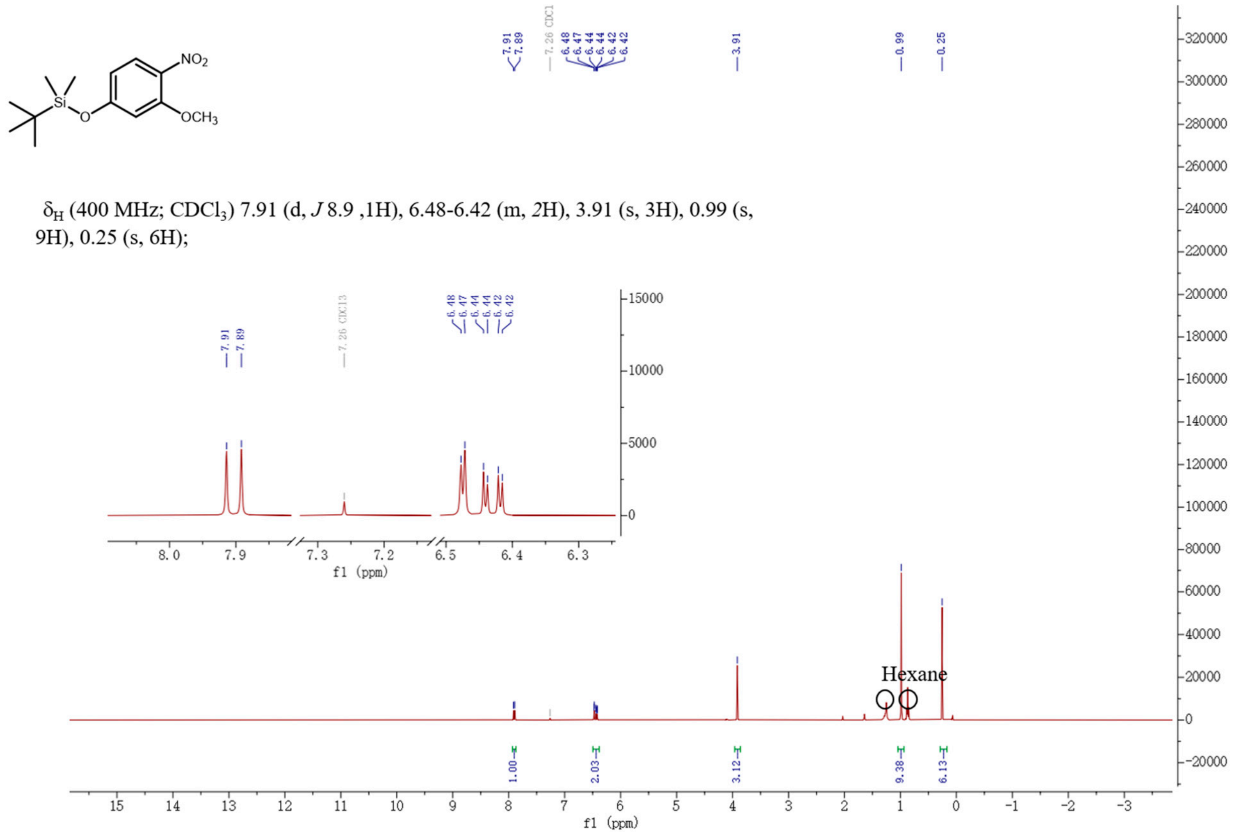

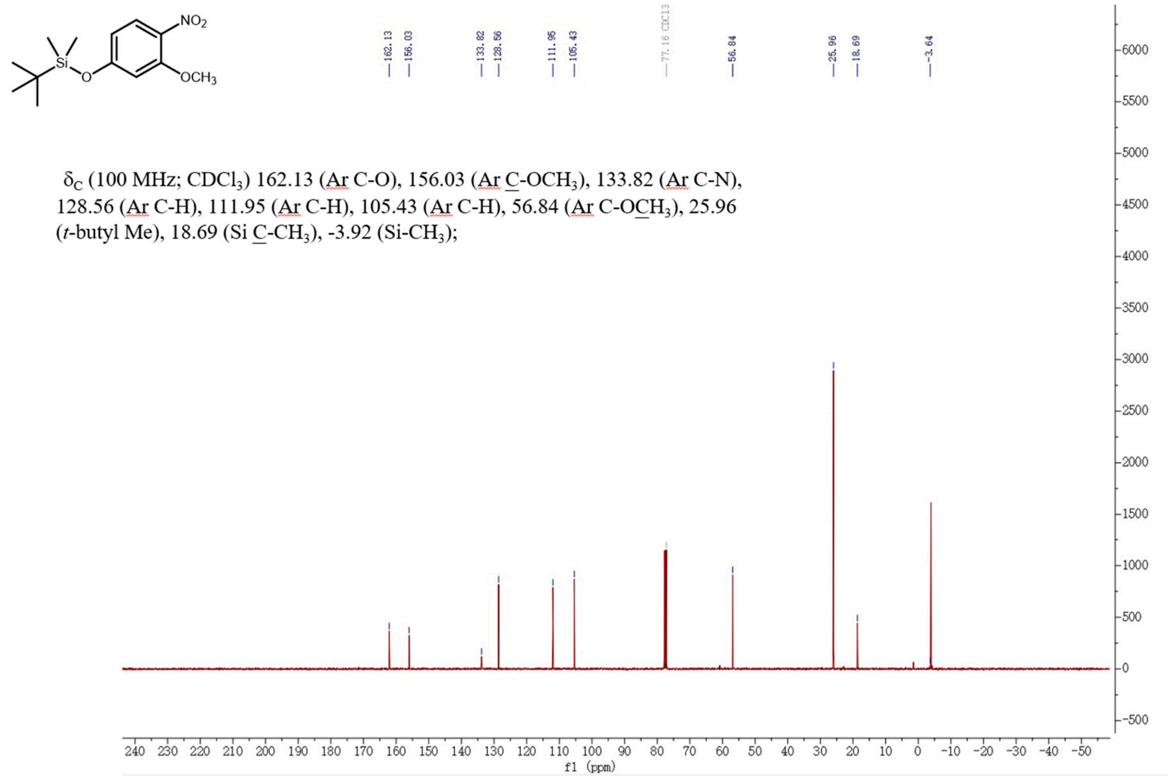

Figure S10. Calibrated NMR spectra for **6** showing ^{1}H (**top**) and ^{13}C (**bottom**) chemical shifts.

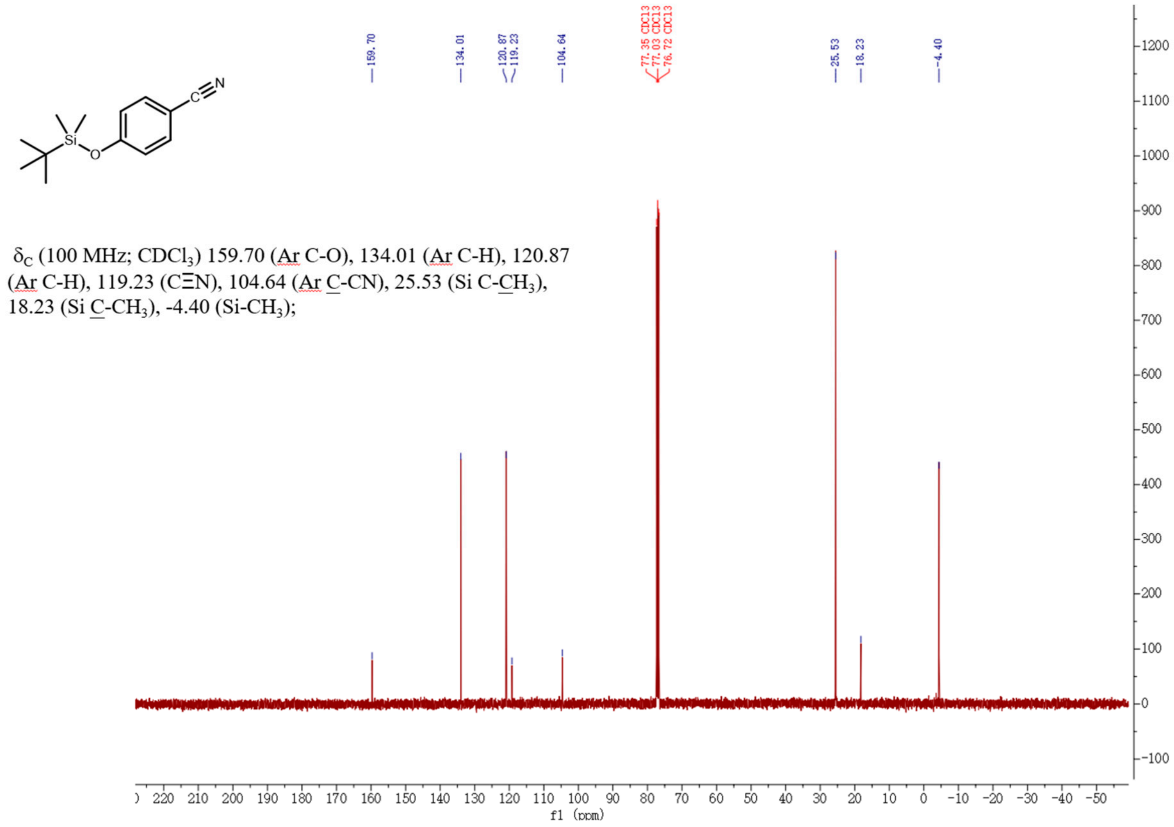

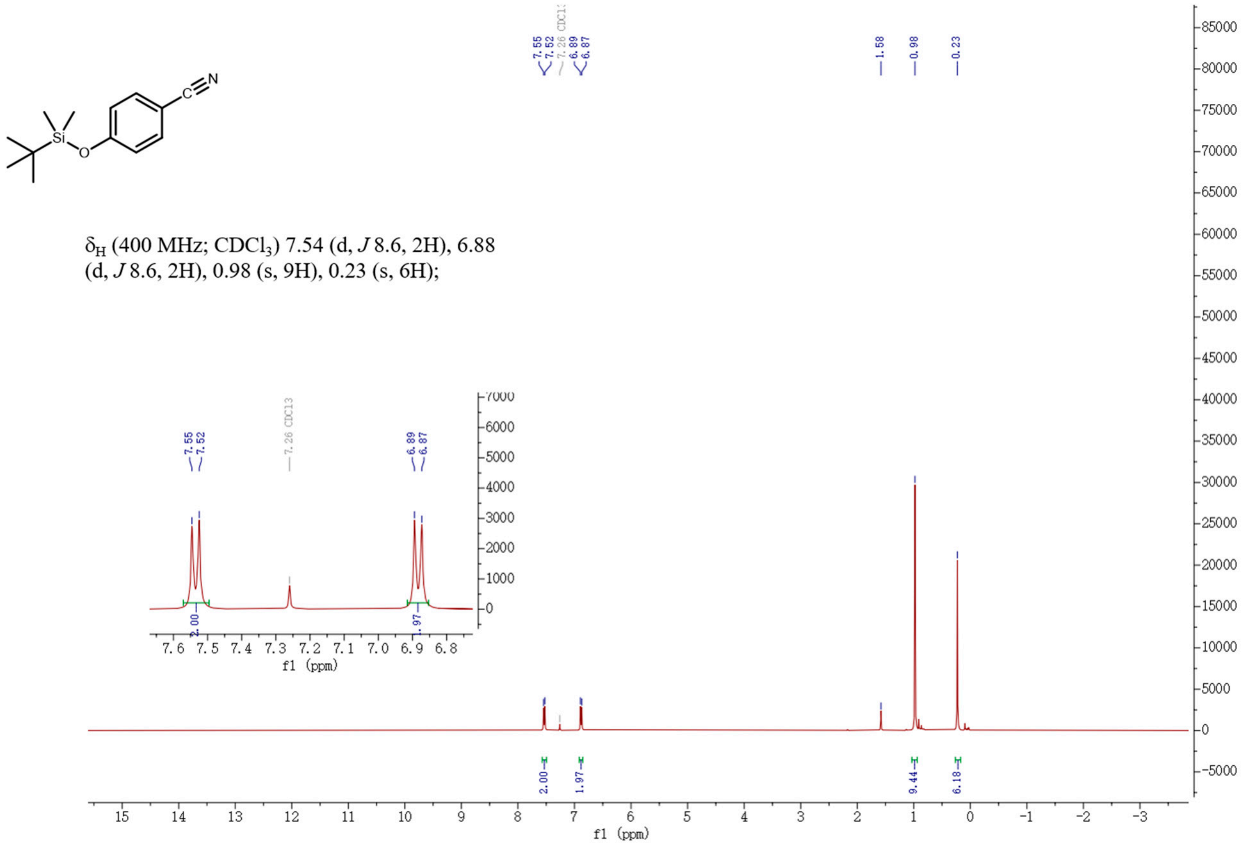

Figure S11. Calibrated NMR spectra for **7** showing ^{1}H (**top**) and ^{13}C (**bottom**) chemical shifts.

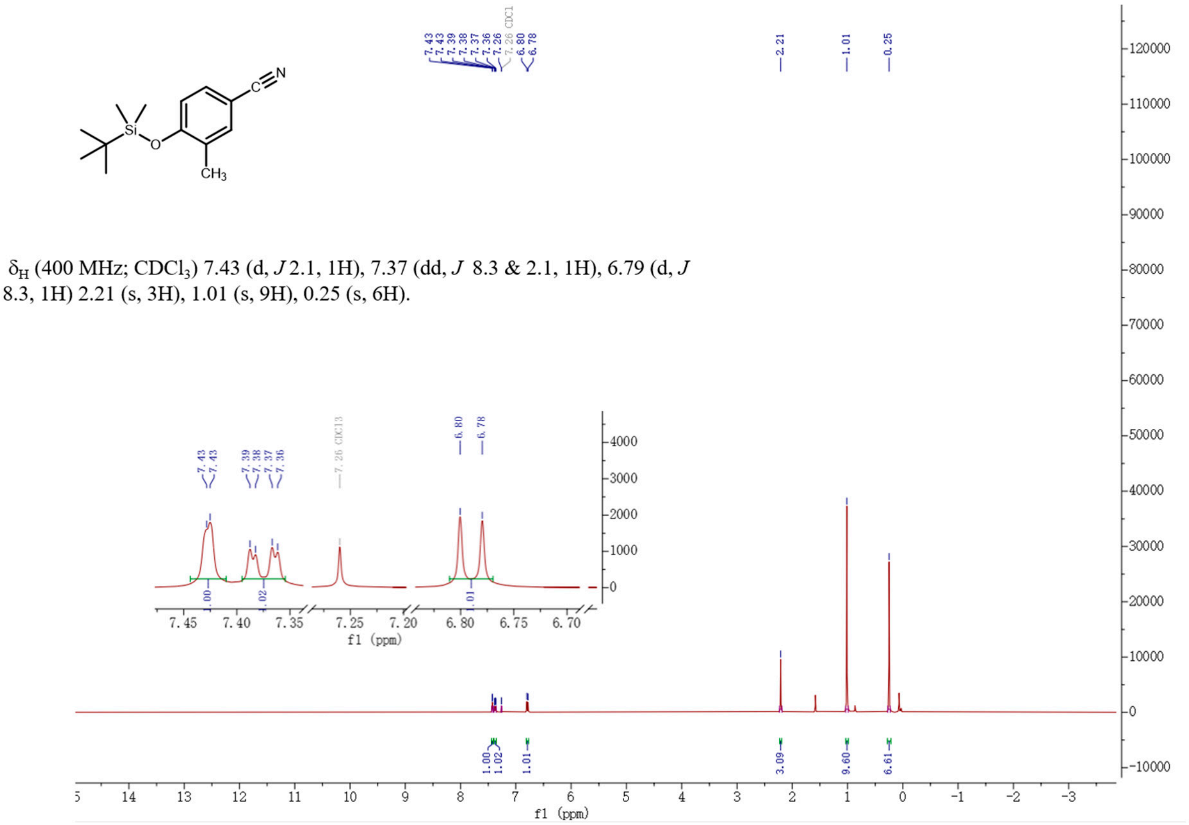

δ_H (400 MHz; $CDCl_3$) 7.43 (d, *J* 2.1, 1H), 7.37 (dd, *J* 8.3 & 2.1, 1H), 6.79 (d, *J* 8.3, 1H) 2.21 (s, 3H), 1.01 (s, 9H), 0.25 (s, 6H).

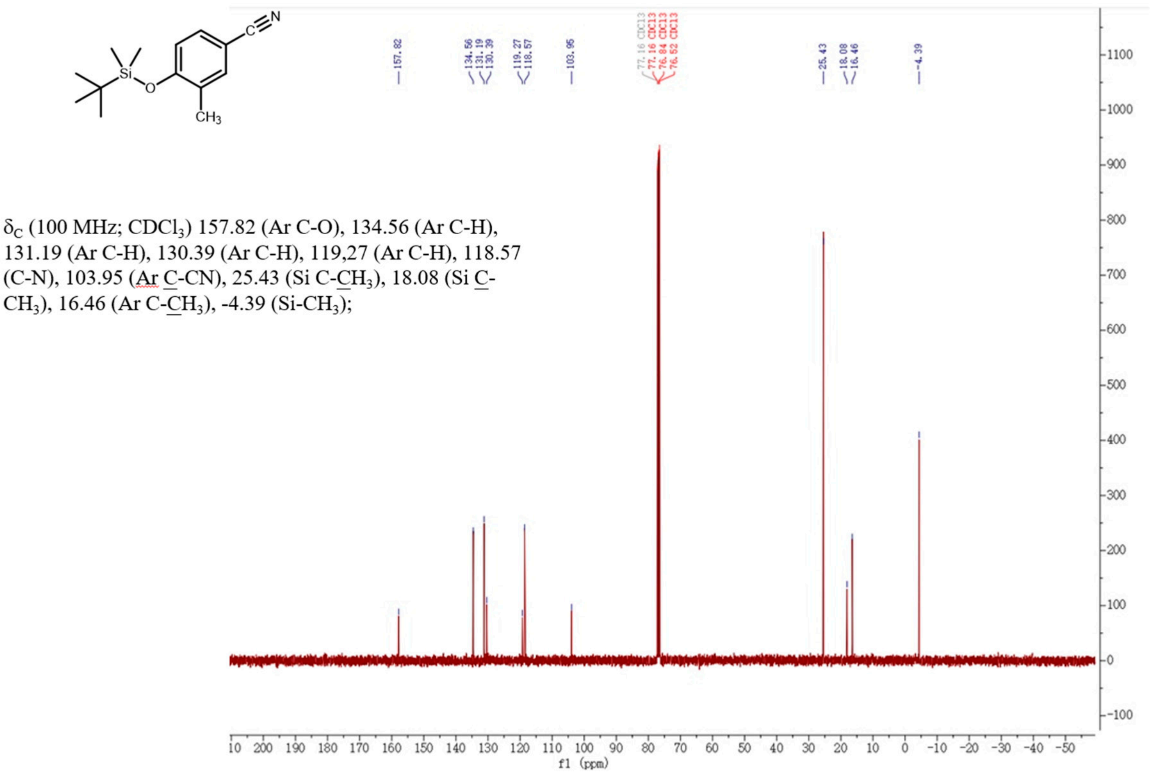

δ_C (100 MHz; $CDCl_3$) 157.82 (Ar C-O), 134.56 (Ar C-H), 131.19 (Ar C-H), 130.39 (Ar C-H), 119,27 (Ar C-H), 118.57 (C-N), 103.95 (Ar C-CN), 25.43 (Si C-CH_3), 18.08 (Si C-CH_3), 16.46 (Ar C-CH_3), -4.39 (Si-CH_3);

Figure S12. Calibrated NMR spectra for **8** showing ^{1}H (**top**) and ^{13}C (**bottom**) chemical shifts.

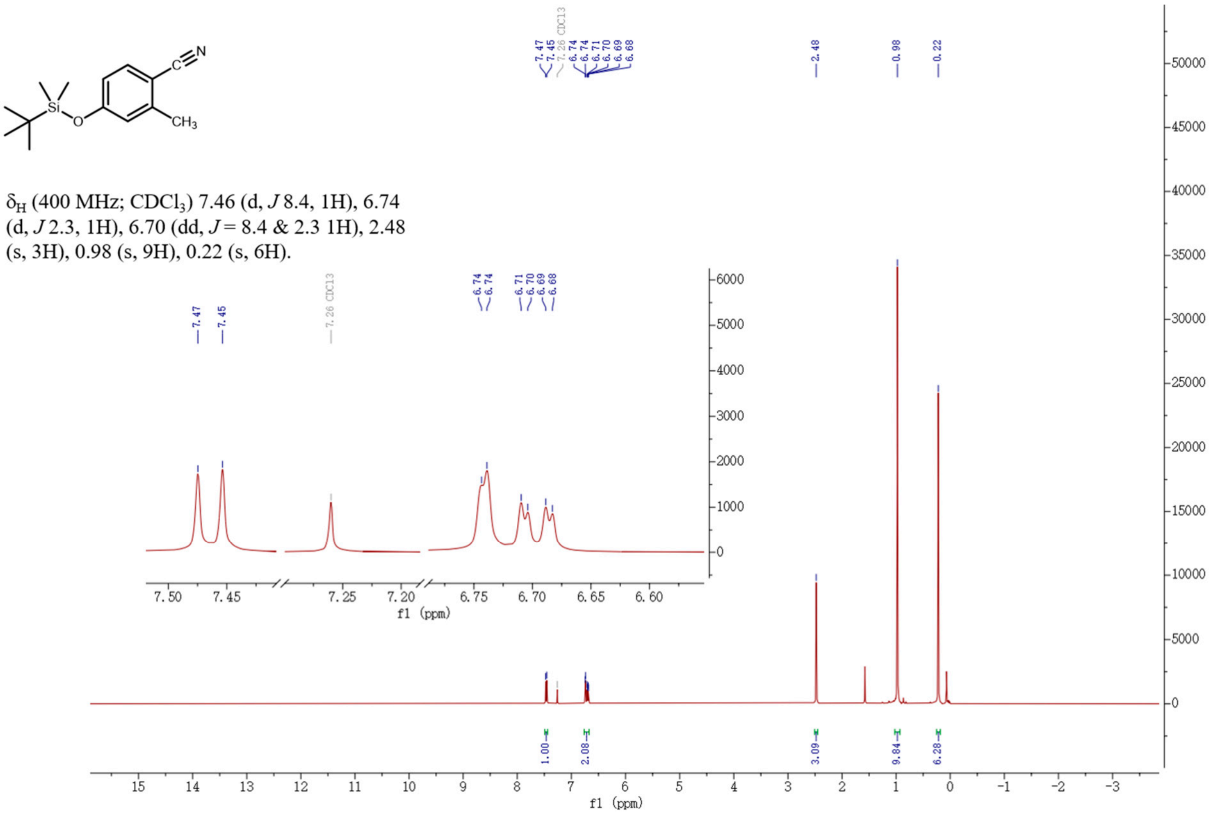

δ$_H$ (400 MHz; $CDCl_3$) 7.46 (d, *J* 8.4, 1H), 6.74 (d, *J* 2.3, 1H), 6.70 (dd, *J* = 8.4 & 2.3 1H), 2.48 (s, 3H), 0.98 (s, 9H), 0.22 (s, 6H).

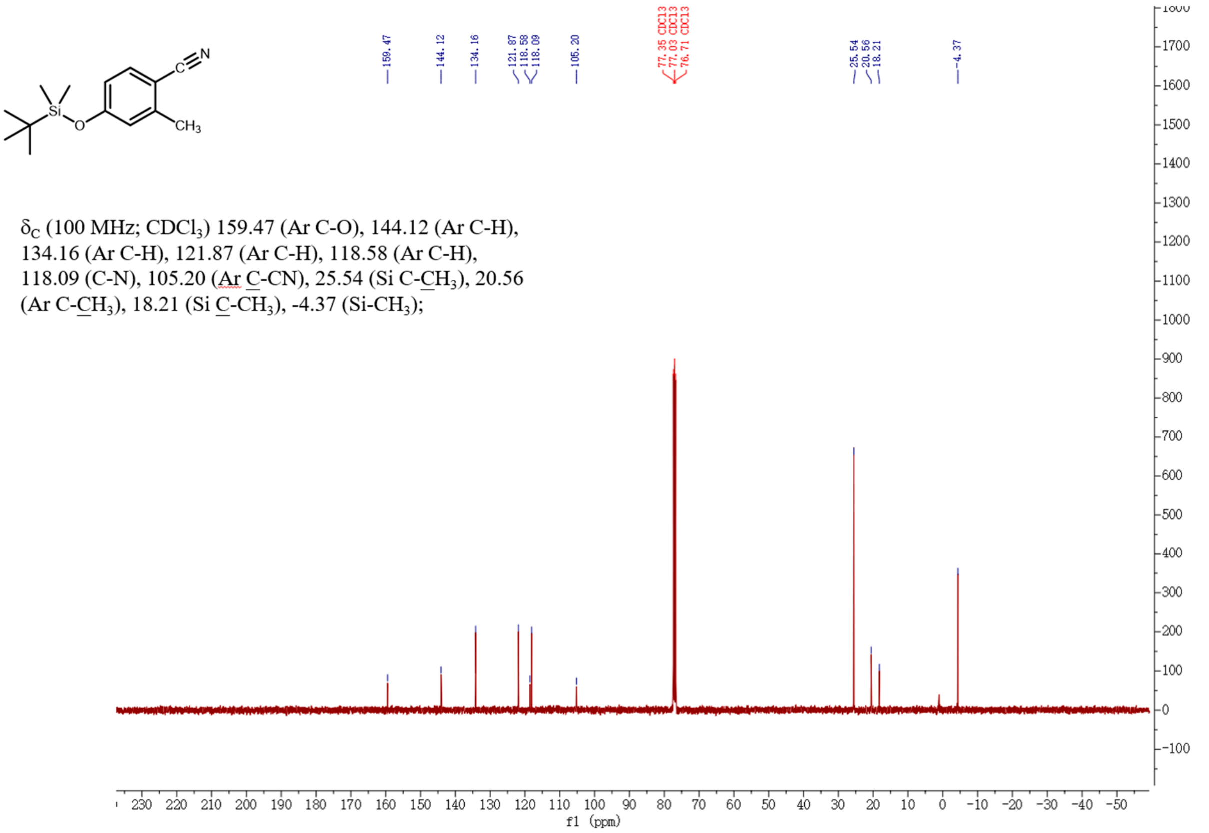

δ$_C$ (100 MHz; $CDCl_3$) 159.47 (Ar C-O), 144.12 (Ar C-H), 134.16 (Ar C-H), 121.87 (Ar C-H), 118.58 (Ar C-H), 118.09 (C-N), 105.20 (Ar C-CN), 25.54 (Si C-CH_3), 20.56 (Ar C-CH_3), 18.21 (Si C-CH_3), -4.37 (Si-CH_3);

Figure S13. Calibrated NMR spectra for **9** showing ^{1}H (**top**) and ^{13}C (**bottom**) chemical shifts.

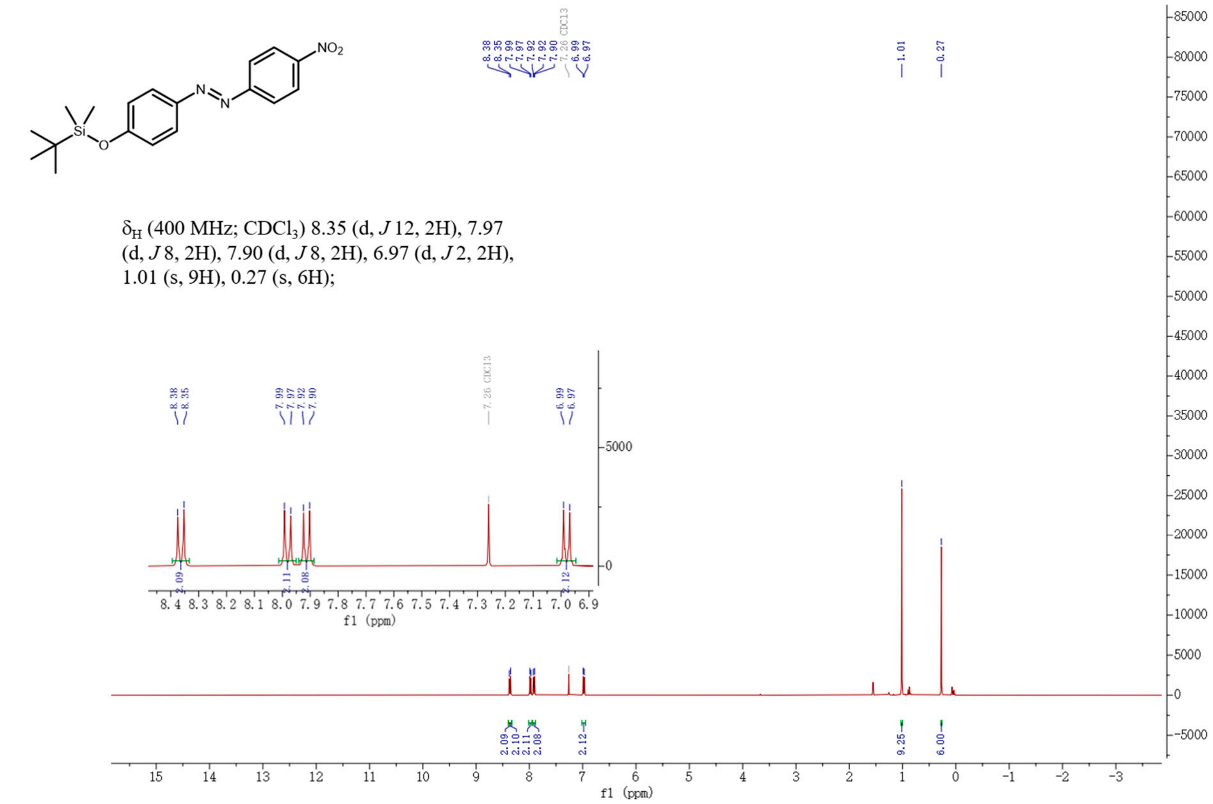

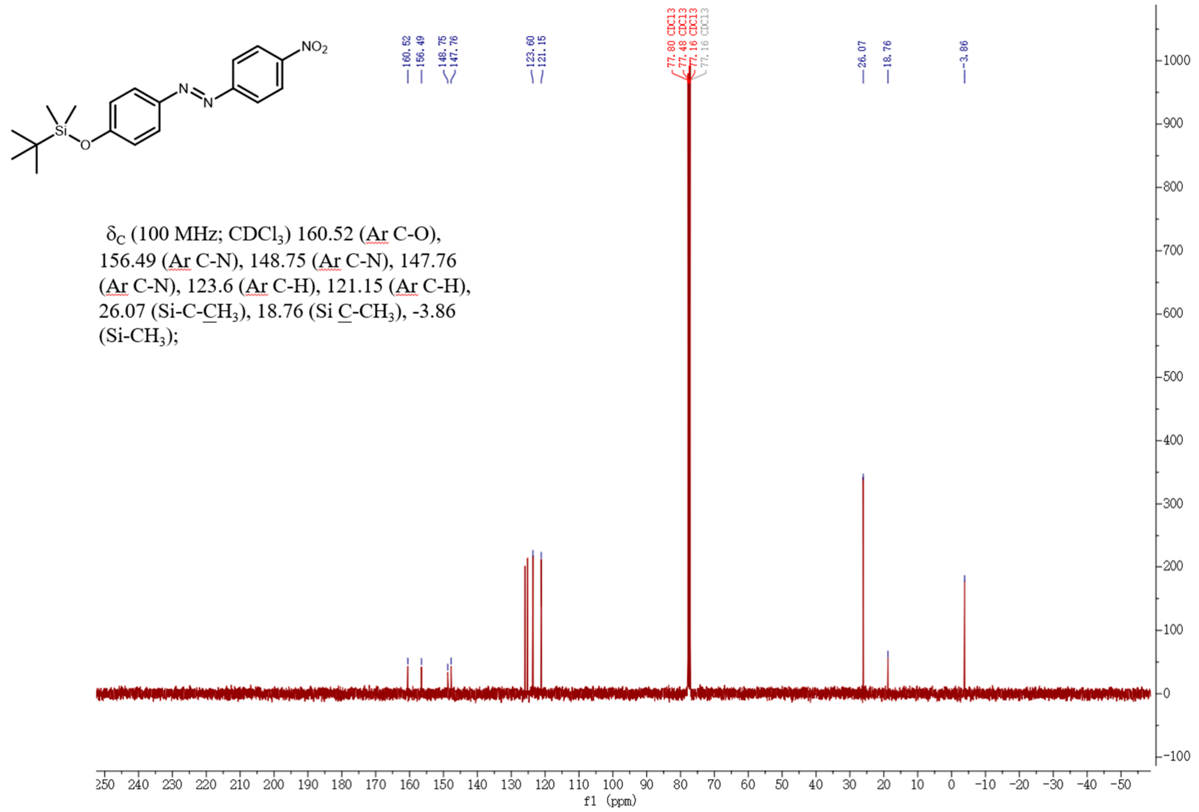

Figure S14. Calibrated NMR spectra for **10** showing ^{1}H (**top**) and ^{13}C (**bottom**) chemical shifts.

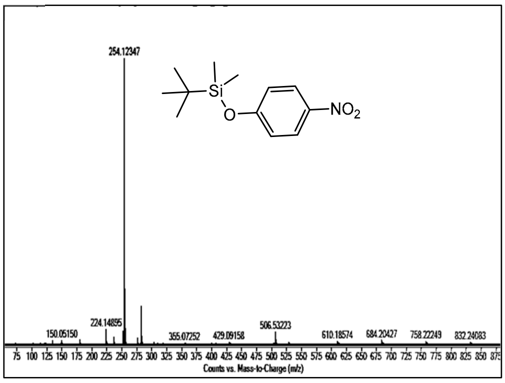

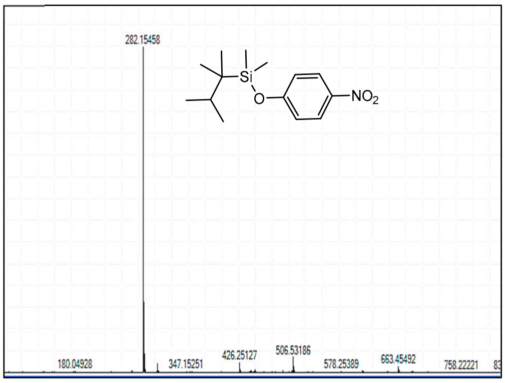

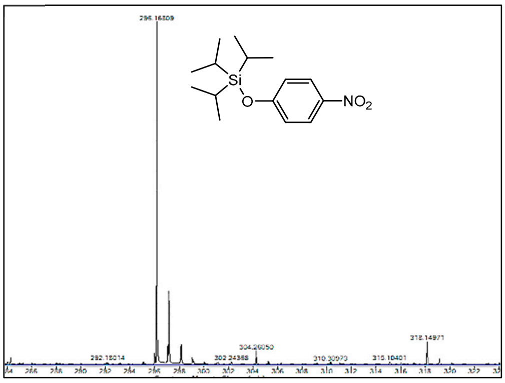

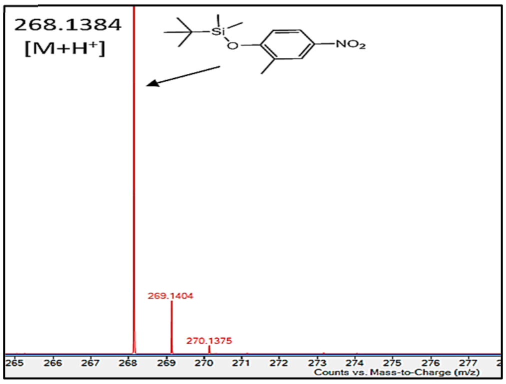

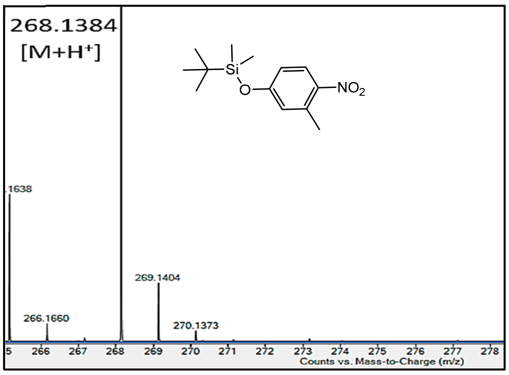

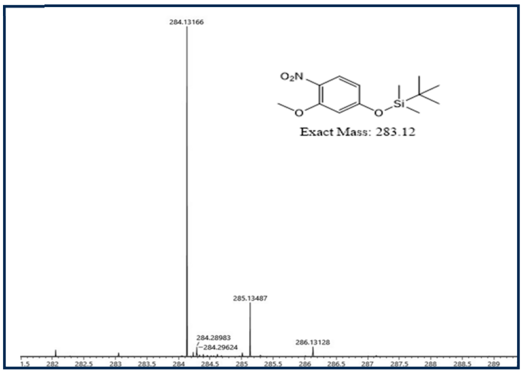

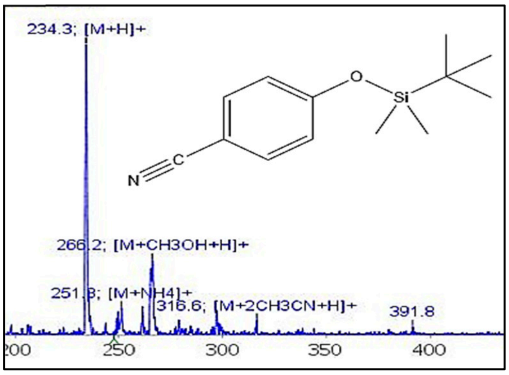

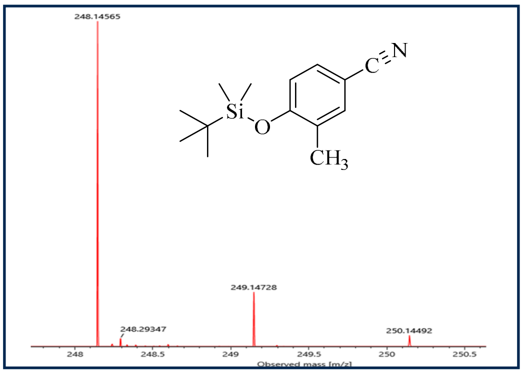

Figure S15. ESI+ mass spectra of substrates **1-10**.

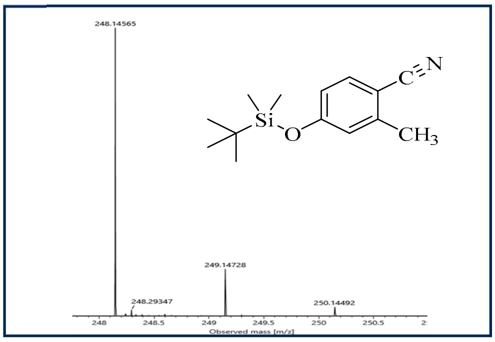

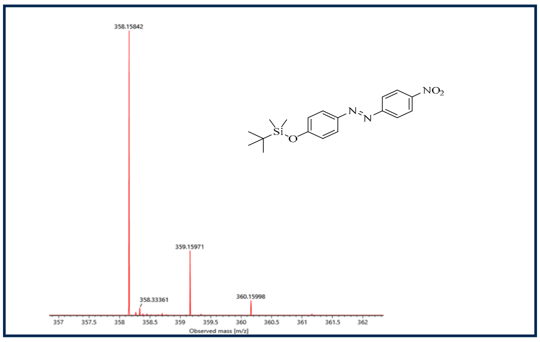

Figure S15 (continued). ESI+ mass spectra of substrates **1-10**.
